# Designing and implementing solution-oriented team science initiatives—a chronic pain example

**DOI:** 10.3389/fpain.2025.1669072

**Published:** 2025-12-16

**Authors:** Nele A. Haelterman, Armen N. Akopian, Kyle D. Allen, Yenisel Cruz-Almeida, Christopher R. Donnelly, Brendan Lee, Rebecca Lenzi, Anne-Marie Malfait, Marie Mancini, Maryann E. Martone, Joost B. Wagenaar, Kim C. Worley, the RE-JOIN Consortium

**Affiliations:** 1 Molecular and Human Genetics Department, Baylor College of Medicine, Houston, TX, United States; 2 Department of Endodontics, School of Dentistry, The University of Texas Health Science Center at San Antonio (UTHSCSA), San Antonio, TX, United States; 3 Department of Orthopaedics and Rehabilitation, University of Florida, Gainesville, FL, United States; 4 Center for Translational Pain Medicine, Department of Anesthesiology, Duke University Medical Center, Durham, NC, United States; 5 National Institute of Arthritis and Musculoskeletal and Skin Diseases, National Institutes of Health, Bethesda, MD, United States; 6 Department of Medicine, Division of Rheumatology, Rush University Medical Center, Chicago, IL, United States; 7 Department of Neuroscience, University of California, San Diego, San Diego, CA, United States; 8 Department of Biostatistics, Epidemiology, and Informatics, University of Pennsylvania, Philadelphia, PA, United States

**Keywords:** team science and practice, chronic pain, joint disease, osteoarthiritis, temporomandibular joint disease, pain

## Abstract

Large, collaborative projects that combine researchers from multiple scientific disciplines have become an integral part of the scientific endeavor. While transdisciplinary team science projects hold great potential, they also come with a unique set of challenges compared to monodisciplinary projects. The Science of Team Science (SciTS) field has developed multiple conceptual models and frameworks that guide the design, implementation, and evaluation of team science initiatives to maximize their potential for making breakthrough discoveries and creating solutions for complex problems. While these conceptual models contain a trove of valuable information for successful team science, guidance on how to effectively implement them is lacking. Here, we describe our experiences with implementing conceptual SciTS models to design and establish the REstoring JOINt health and function to reduce pain (RE-JOIN) consortium, a transdisciplinary team science project aimed at elucidating the mechanisms underlying chronic joint pain, with a solution-oriented focus. We highlight our experiences and challenges with implementing existing conceptual models and provide practical tips and guidance for designing and implementing solution-oriented team science initiatives.

## 1 Introduction

Transdisciplinary team science projects have become an increasingly common and effective strategy to tackle complex public health problems (1, 2). Teams containing researchers from distinct backgrounds, crossing scientific disciplines, institutions, or in some cases national boundaries, unite to provide their unique perspective on a shared scientific problem, with the overarching goal to advance scientific knowledge by integrating existing or new knowledge, technologies, and/or resources to establish innovative frameworks that provide new insights into a scientific problem (3, 4). Transdisciplinary team science projects have unique challenges compared to monodisciplinary research projects as they aim to leverage individual differences among team members to enhance the breadth and depth of insight and innovation (3, 5).

The science of team science (SciTS) field emerged to better understand the factors that facilitate or impede effective team-based research, with the goal of providing solutions that help overcome its many challenges (4, 6). By integrating qualitative and quantitative research methods, SciTS develops evidence-based best practices and conceptual models for effective team-based research in a broad range of scientific disciplines, including translational research, which have been recently described in great detail (4, 5, 7–10). Briefly, setting team science projects up for success requires developing and nurturing a collaborative mindset and trust between researchers while simultaneously developing a shared language that overcomes domain-specific communication barriers along with a set of clearly defined shared scientific goals to work towards (8, 11–13). To achieve this, the team requires specialized infrastructure that supports effective communication, data and file sharing, and coordination across its disciplines and members (12). Hence, forging effective transdisciplinary collaboration is a formidable challenge that requires a vast supportive framework and a carefully laid out plan to accomplish.

**Figure 1:**
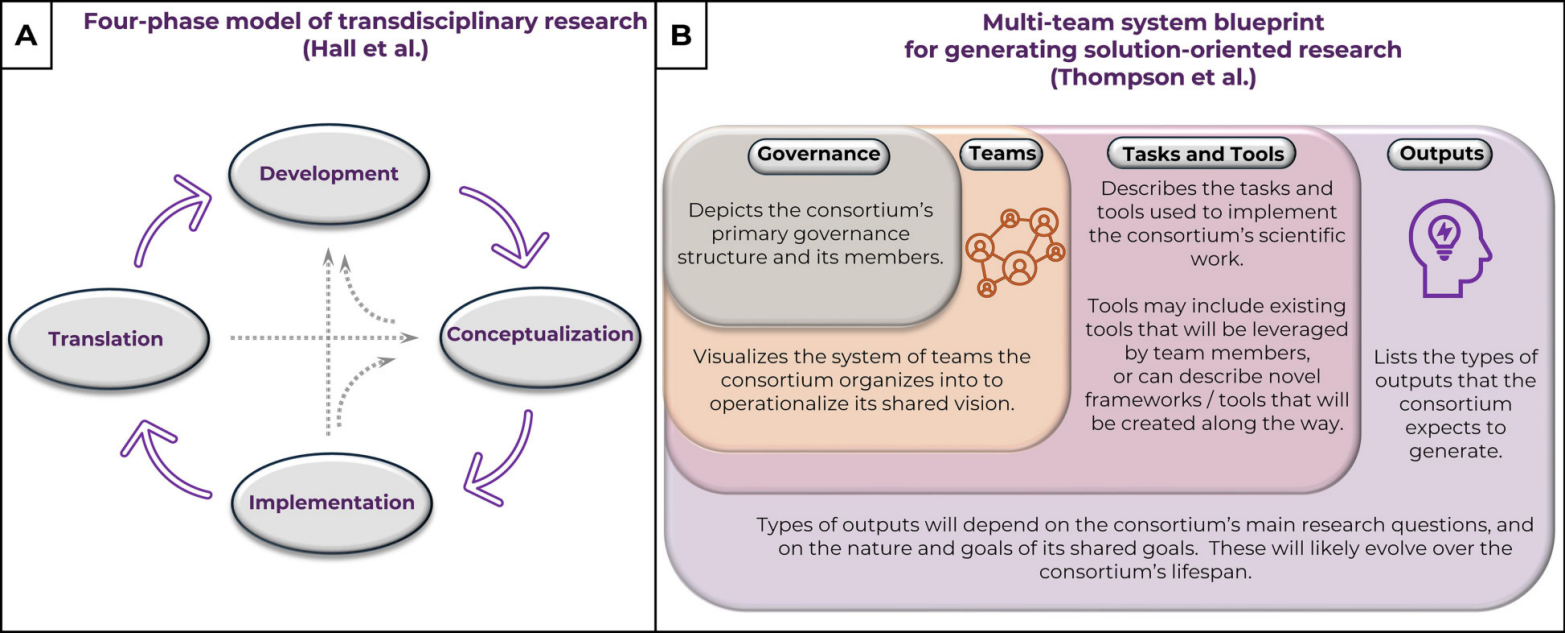
Evidence-based models and frameworks for team science, developed by the SciTS field. **(A)** Hall et al.'s four-phase model of transdisciplinary research describes the lifecycle of team science projects (14). **(B)** Thompson et al.'s multi-team system blueprint for generating solution-oriented research provides budding consortia with the resources to develop a “master plan” detailing how the consortium plans to operationalize its shared goals and reach them (18).

In 2012, Hall et al. introduced a four-phase model to describe a team science project's evolution (Figure 1A), beginning with the identification or definition of the team's central scientific or societal problem and its members (*development phase*), to developing its research questions, hypotheses, conceptual models, or study designs that will address the problem (*conceptualization phase*), to then executing the planned research studies (*implementation phase*), and finally analyzing obtained results using transdisciplinary approaches to create innovative strategies to overcome or improve the central problem (*translation phase*) (14). Several additional conceptual models have since been developed by SciTS researchers that offer insights into evaluating or enhancing collaborative processes (15–17). In addition, Thompson et al. recently described novel conceptual models to guide the design, implementation, and evaluation of transdisciplinary team science projects (18). Thompson's blueprint model for generating solution-oriented research provides a framework for multi-team systems to think through (1) their organizational structure, (2) the tasks that will help them reach their shared goals, (3) the tools they will use or develop along the way, and (4) the outputs they plan to generate (Figure 1B). While these models and frameworks are incredibly helpful, there is a need for guidance on how to implement these strategies, or on how to tackle the challenges that arise as part of this process. This is particularly true for the pain and addiction fields, which have seen an explosive growth in transdisciplinary team science in recent years (19–21).

Here, we build on the knowledge and guidance provided by the SciTS field in the context of cardiovascular, cancer, computational research and other fields (10, 17, 18, 22, 23) and integrate our experiences to outline guidance on establishing effective team science initiatives. Specifically, we use Hall and Thompson's conceptual models to describe the steps we took and the infrastructure we developed while establishing the REstoring JOINt health and function to reduce pain (RE-JOIN) Consortium, a transdisciplinary effort to improve our understanding of chronic joint pain (14). We highlight challenges faced along the way and the solutions that we developed to overcome them. Building on the SciTS literature, RE-JOIN's use case scenario outlines a roadmap for building the tools, resources, and team dynamics needed for successful and effective transdisciplinary team science projects in the field of joint pain research and beyond.

## 2 How and where to start: considerations when embarking on team science projects

At the onset of a large team science project, it is vital to develop a supportive framework to bolster collaboration and empower the group's researchers to focus on science (8, 14, 18, 23). Such frameworks include clearly defined collaborative goals, an effective operational and organizational structure, a feeling of trust among team members, integration platforms for data and resources, project-specific harmonization elements, and others. Each of these components takes time to develop and piece together into the framework that will best support the project. Importantly, the maintenance of this framework is a highly dynamic process, as many aspects will evolve throughout the project's lifespan depending on the team's needs and aspirations.

Based on our experiences with team science endeavors, such as RE-JOIN, here are some key steps to take during the various phases of the consortium's lifecycle, as described by Hall et al. (Figure 1A) (14):

– *Development phase*: This is when team members first get to know one another and learn about their values, skills, and research priorities. To enable this process, it is important to establish an effective, transparent communication process, and to start building reliable, accessible information and knowledge management workflows. This phase also includes actions needed to foster a collaborative mindset, such as establishing transparent governance and decision-making processes. During this and the next phase, it is helpful to think through and complete Thompson et al.'s conceptual model for designing and implementing transdisciplinary team science projects (Figure 1B) (18). Completing this blueprint will guide the team through aligning and integrating individual research priorities into a collaborative research plan and will enable the team to identify the most effective way to implement this plan.
– *Conceptualization phase*: During this phase, the team starts aligning their individual goals and research plans into a shared vision. Here, it is important to develop a shared or common language that uses similar vocabulary and permits effective communication across the project's scientific disciplines. This will in turn facilitate discussions around experimental and data harmonization. In addition, the team will spend time developing and harmonizing the methods and tools needed to achieve the identified shared goals. This is typically achieved in working groups or task forces, where team members pursue specific tasks or goals. As the team's research groups will likely start data collection during this phase, it is beneficial to test and streamline data and method sharing pipelines for various data types and technologies. This can be done with a pre-existing or a pilot data set that is not planned to directly support a manuscript, for example a data set generated during the method optimization process. Discussing experiences with initial data and/or method sharing workflows can take place during task-oriented working group meetings, as they will facilitate discussions around method optimization and will accelerate establishing a standardized and harmonized minimal data set. Towards the end of the conceptualization phase, an evaluation moment can be built in to allow members to voice their opinion on how operational and collaborative workflows can be improved.
– *Implementation phase*: As decentralized research groups collect, share, and analyze data at scale during this phase, some issues or needs will inevitably come to light, revealing needs for further refinement of the data sharing process and/or the consortium's minimal data standard. This can in turn lead to the development of novel frameworks, analytical, or conceptual models that will likely accelerate research for the broader scientific community.
– *Translational phase*: During the project's final phase, integrated analyses of the team's harmonized datasets will yield novel, intriguing conclusions and spark novel research questions. This, in turn, sets the stage for the team to take the next step: revisiting its collaborative research goals and team membership to address the next big scientific challenge.

## 3 Putting SciTS models in practice—a chronic joint pain example

Joint-disease—associated chronic pain is a significant worldwide health problem for which few effective treatments exist. For example, osteoarthritis affects nearly 8% of the global population, whereas ∼5% of the US population has pain related to temporomandibular joint disease (TMD) (24, 25). To improve care for patients with joint pain, novel therapies are needed that specifically target the mechanisms that transmit joint pain. However, progress on this front is hampered by a limited understanding of the neuronal connectivity patterns that mediate pain sensation in these tissues. Improving our understanding of the cellular and molecular changes that cause and mediate pain sensation in the joint will require experts from various scientific backgrounds to come together and develop novel technologies to label, image, and characterize joint-innervating neurons in human and animal models of joint disease.

**Figure 2:**
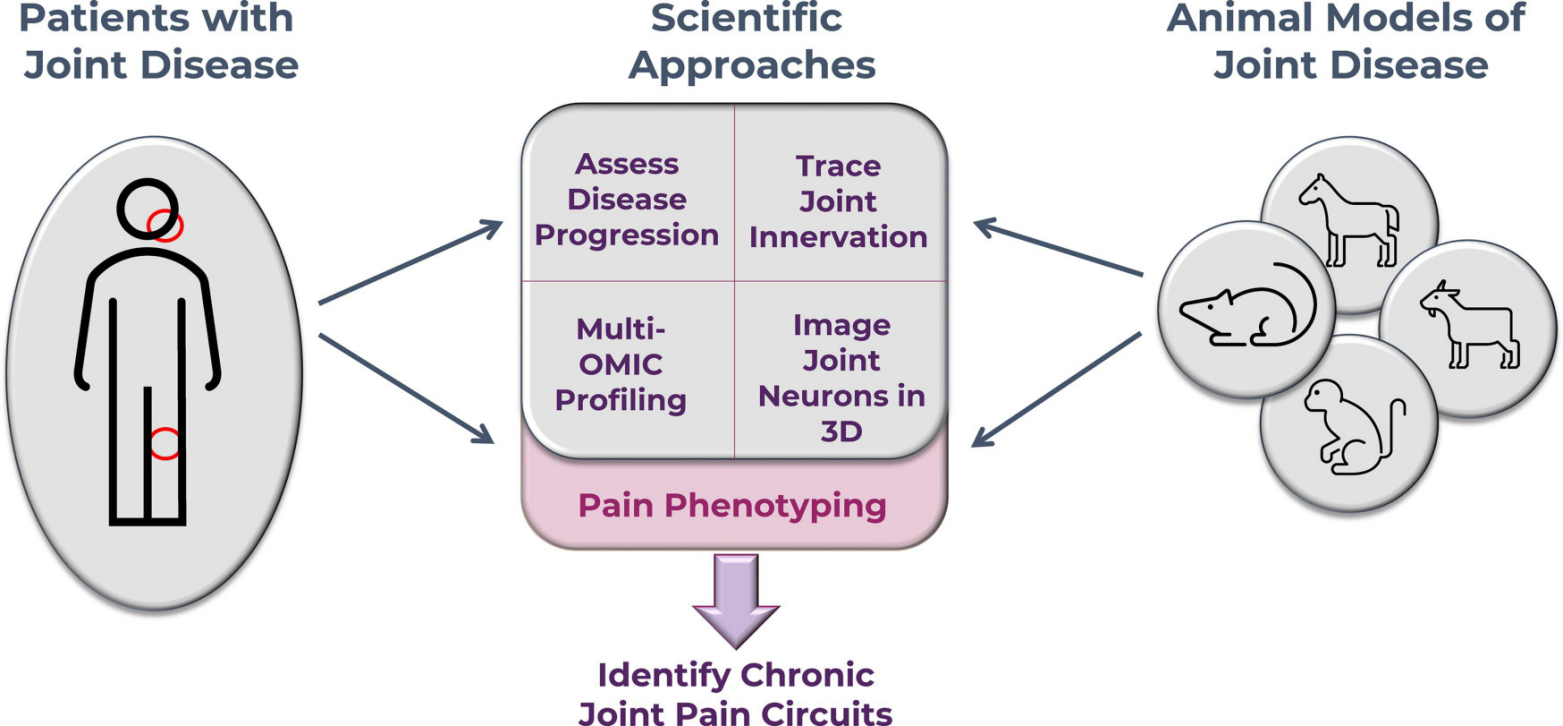
RE-JOIN's multidisciplinary approach combines pain phenotyping, neuronal labeling, molecular and cellular profiling, histopathological, and therapeutic approaches to identify drivers of chronic joint pain.

In November 2022, the National Institutes of Health (NIH) and the Helping to End Addiction Long-term® (HEAL®) Initiative established the RE-JOIN Consortium with the goal of better understanding the molecular and cellular roots of joint pain. Through transdisciplinary team science, RE-JOIN will study the factors that determine joint health and pain in diverse animal models of joint disease—including rodent, primate, and equine models—and will correlate them with clinical findings obtained from patients with joint disease (Figure 2). To do so, RE-JOIN'ers will develop 3D sensory and autonomic innervation maps of healthy joints, and will characterize how these maps change to mediate joint pain in the context of joint disease, age, biological sex, exercise, and treatment. As a first step towards this goal, RE-JOIN will focus on two joints—the knee and temporomandibular joint (TMJ)—with the expectation that developing methods for these joints can be extrapolated to other joints. Next, consortium members will develop the data, analytical pipelines, and frameworks needed for integrative analysis of its different data types (imaging, omics, behavior, etc). Ultimately, RE-JOIN's overarching objective is to provide the community with a rich anatomical and molecular resource of curated databases for studying mechanisms underlying joint pain. This, in turn, will serve as the starting point to identify potential therapeutic candidates that may improve joint health or transform joint pain treatment.

### 3.1 Establish a transdisciplinary team

Transdisciplinary teams are diverse by nature, including researchers from various disciplines, project managers and data scientists, knowledge management professionals, as well as community, agency, and policy stakeholders. This combination of expertise, personalities, and skills creates opportunities to innovate and advance knowledge with a lasting impact, so long as the group's efforts are coordinated, and its research goals are aligned. While the composition of an individual principal investigator's team is typically determined by the investigator themselves, that of a team science project often forms organically based on the research goals of the group. In addition, depending on the project's nature and structure, the team's composition may be determined by the project's funding sources. Therefore, while the group's composition may be beyond any member's control, the essential action is to turn the group into a team. This process cannot be rushed and involves several elements, including getting to know each other, establishing a culture of trust, developing a shared language, and developing a team climate. In the resulting team, each member should feel valued and should understand the team's goals, the scientific and practical reasoning behind each goal and their own role(s) in achieving them (26).

**Figure 3:**
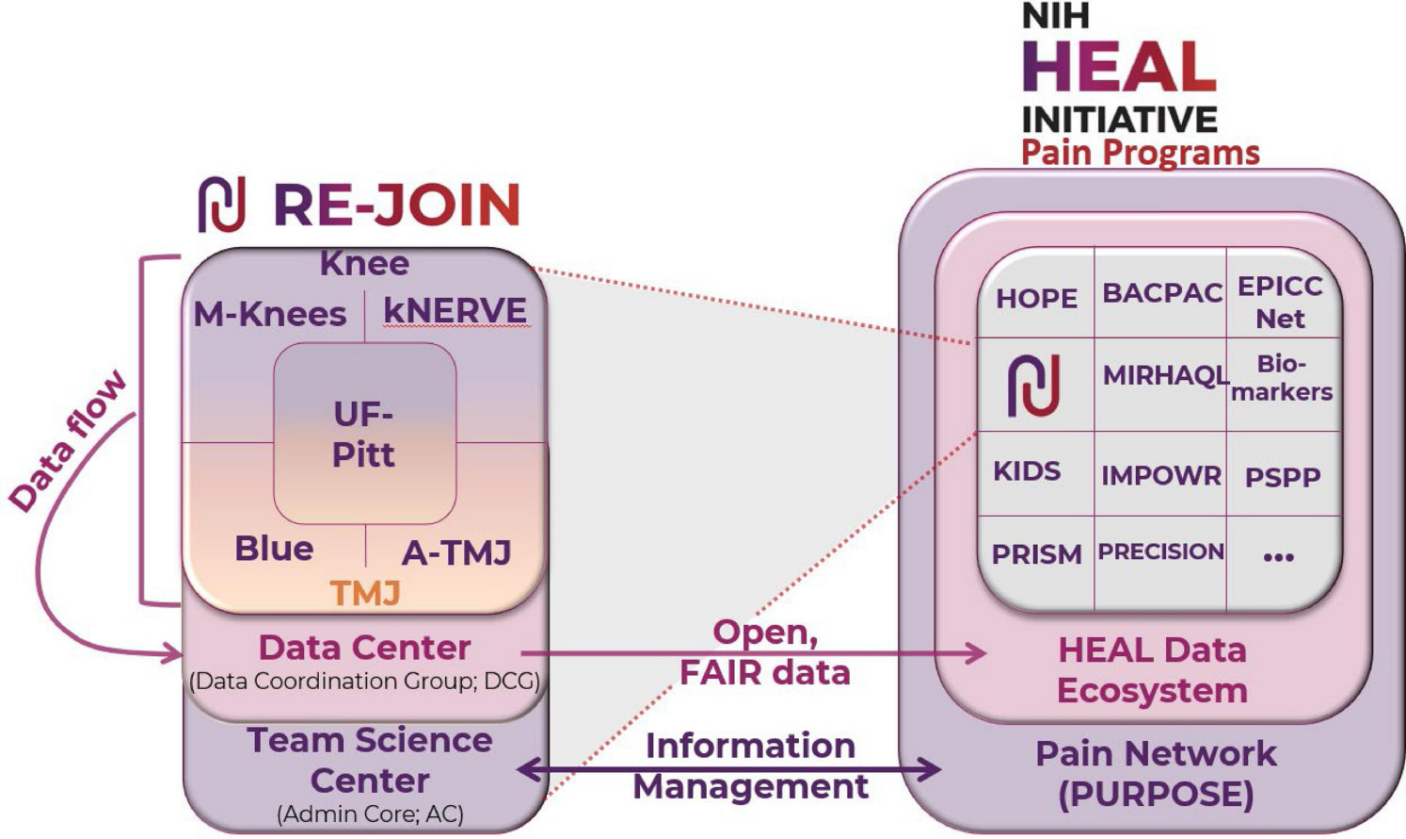
Overview of the RE-JOIN consortium and its member groups as part of the NIH HEAL Initiative. RE-JOIN’s research teams include 2 teams studying knee osteoarthritis (M-Knees, kNERVE), 2 teams that study Temporomandibular (TMJ) disorders (Blue, A-TMJ), and 1 team that investigates both joint diseases (UF-Pitt). Generated harmonized datasets are curated and shared through the data center, while the team science center oversees the consortium’s organizational, structural, collaborative, and information management processes.

In the case of RE-JOIN, 7 teams, spanning 19 disciplines and 20 academic research institutions (Supplementary Table S1), came together to better understand chronic joint pain. RE-JOIN comprises 2 research teams that focus on knee joint innervation (kNERVE, M-Knees), 2 research teams that study temporomandibular joint (TMJ) innervation (A-TMJ, Blue), and one that studies both joints (UF-Pitt) (Figure 3). This organizational design combines the well-established knee osteoarthritis field and the developing TMJ disease field, with the goal of leveraging team science to build upon each field's strengths and overcome its weaknesses. Each multi-site, multi-project research team consists of 25 to 30 topic experts from various disciplines, resulting in roughly 130 RE-JOIN'ers with expertise in joint diseases, pain, neural circuit mapping, tissue clearing, multi-omics, and others (Supplementary Table S1). In addition, RE-JOIN is strengthened by the project managers, curators, infrastructure engineers, data and team scientists at the consortium's team science center (admin core, AC) and data coordination center (DCG), as well as project scientists and program officers from several institutes at the NIH, including the National Institute of Arthritis and Musculoskeletal Research (NIAMS), the National Institute of Neurological Disorders and Stroke (NINDS), and others (Figure 3). Hence, this group of experts contains the know-how to form a transdisciplinary team that can cross individual disciplinary boundaries to create the novel framework needed to understand the biological underpinnings of joint pain.

Finally, maintaining a team climate that bolsters collaboration and innovation over the course of the project will be an ongoing process because the team's research goals will evolve, team dynamics may shift, and some personnel will inevitably turn over. Therefore, it is important for consortium leadership to regularly assess whether its working groups and committees contain representatives from all relevant disciplines and subgroups, and that all members feel included and valued. This can be achieved through surveys or open group discussions.

### 3.2 Design a multi-team system for solution-oriented team science

**Figure 4:**
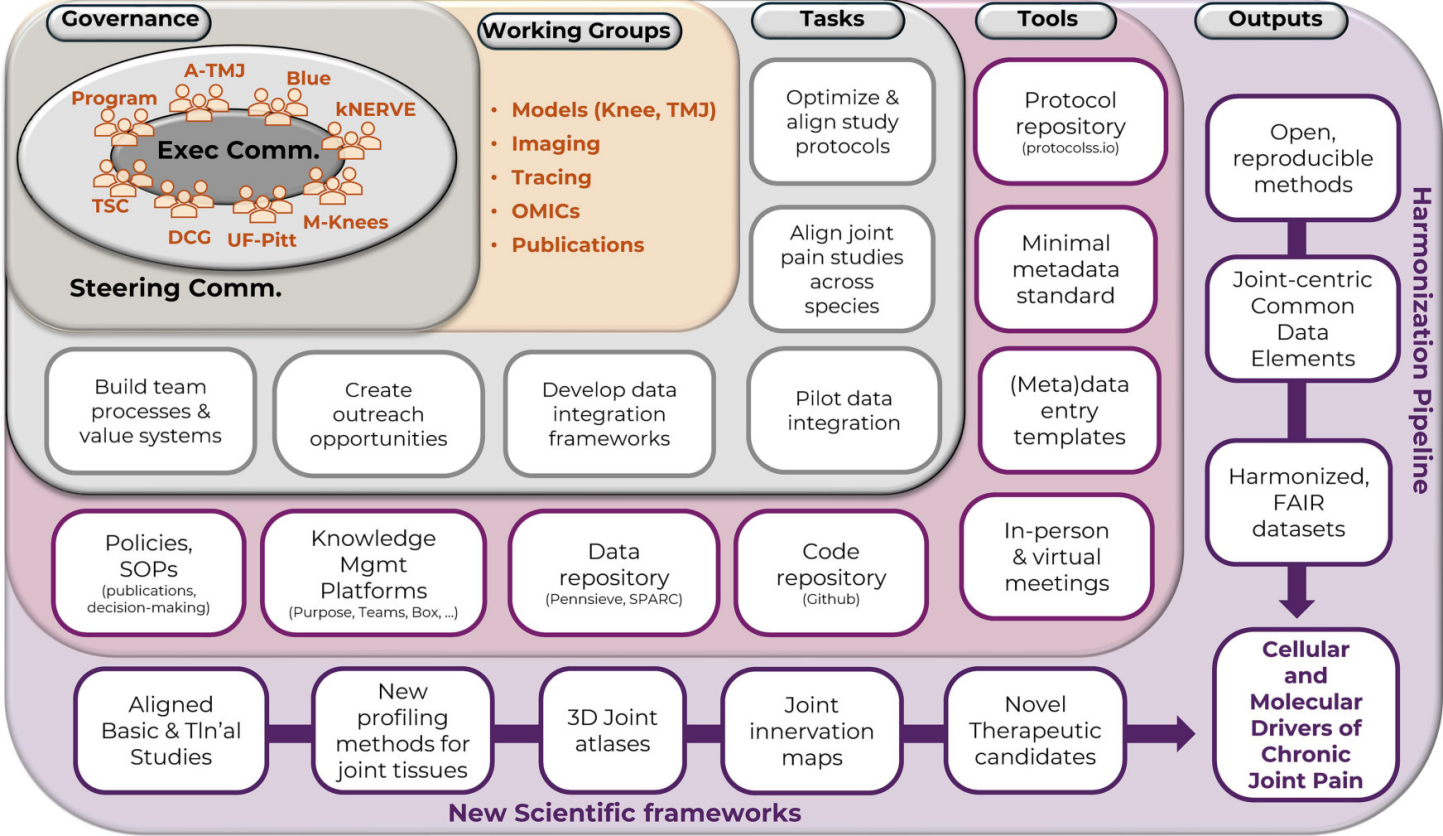
RE-JOIN's blueprint for solution-oriented research, modified from Thompson et al's conceptual model (18).

Team Science's potential can be unlocked by aligning a team's shared vision with the complex, interdependent tasks (i.e., identifying and creating the methods, data, tools, workflows, etc.) needed to provide insights and innovative solutions for its scientific problem(s) (8, 15). Thompson's “blueprint for generating solution-oriented research” provides a framework for designing and implementing transdisciplinary team science initiatives with a solution-oriented focus (18). This framework is organized in 5 team dimensions (governance, teams/working groups, tasks, tools, and outputs) and is designed to align a team's outputs with its shared goals. Below, we describe how RE-JOIN leveraged this blueprint to guide our collaborative efforts and identify the tasks and outputs to align with our common goal: identifying cellular and molecular drivers of chronic joint pain (Figure 4).

– *Governance*: this dimension highlights the consortium's primary governance structure that oversees its scientific direction and makes key operational decisions (18). RE-JOIN's governance is overseen by its steering committee, which includes all members from its 5 research subgroups (A-TMJ, Blue, kNERVE, M-Knees, UF-Pitt), program, team science center (TSC), and data coordination group (DCG) (Figures 3, 4).
– *Working groups*: team members organize into working groups (WG), or task forces, which form the second dimension of Thompson's model. These groups are responsible for creating and implementing the necessary elements for achieving the team's shared goals. In the case of RE-JOIN, the nature of the WGs was defined to align with our shared research goals, which center around developing and harmonizing novel methods, frameworks, and technologies for labeling and profiling joint-innervating neurons to characterize the molecular and cellular drivers of chronic joint pain by (See Step 3: “**Define Collaborative Goals**”).
– *Tasks*: This dimension represents actionable steps to achieve the consortium's shared goals. Tasks are typically defined by the team's governing body, and are carried out by its WGs or task forces. Several tasks will apply to most team science initiatives (establish team processes and value systems, develop data harmonization and integration frameworks), while others represent project-specific tasks, such as aligning pain studies across studies for RE-JOIN (Figure 4). Of note, these tasks will also include creating novel frameworks (outputs) needed to achieve shared goals. For example, RE-JOIN members quickly realized that integrating distinct data types related to joint innervation (imaging, behavioral, molecular data, etc) would require a detailed anatomical 3D atlas of the joint and the neuronal tissues innervating them. This (joint atlas), and other novel frameworks are listed as outputs (Figure 4); they represent shared RE-JOIN goals with multiple specific tasks to accomplish them.
– *Tools*: This level includes the tools used by team members and working groups for accomplishing their tasks. In RE-JOIN's case, these included platforms for knowledge and information management (Purpose, Teams, Box, etc), and FAIR repositories for sharing data (Pennsieve, SPARC), methods (protocols.io), and code (Github) (Figure 4). We also developed 2 novel tools through early tasks related to developing data integration frameworks. RE-JOIN's (meta)data entry templates facilitate data entry for consortium members, ensuring compliance with the consortium's minimal metadata standard and common data elements (27–29).
– Outputs: consortium outputs include interdependent scientific frameworks and data pipelines that build on each other to produce new knowledge, datasets, tools, and frameworks that, in the case of RE-JOIN, will yield novel insights into the molecular and cellular drivers of chronic joint pain and that will inform future programs, policies, and practices (18).

In our experience, identifying shared goal-focused tasks, tools, and outputs is a dynamic, iterative process, as novel needs will emerge throughout the project's lifespan. We found that regular brainstorming in small (working group or task force) and large (steering committee) group settings helped identify emerging needs, tools, and tasks. Follow-up discussions were then held to align these elements with the consortium's overarching goals and to create action plans with clearly defined roles and responsibilities for team members. Such discussions identified the need for several novel scientific frameworks that would be needed to generate and accurately integrate the multi-modal data generated in the various joint disease models studied by the consortium's research (Figure 4). For example, early discussions identified the need for a common coordinate framework of the knee and TMJ joint across species (3D joint atlases) to integrate 3D imaging datasets, and the consortium next created a task force responsible for (1) reviewing existing frameworks, (2) defining team-specific needs for this framework, (3) identifying the tasks, tools, and datasets required for building it and (4) developing an action plan for developing this novel framework. In contrast, later discussions in the OMICs working group (WG) revealed a similar need to develop a 3D atlas of the dorsal root and sympathetic ganglia that contain joint-innervating neuronal cell bodies. This need emerged from the completion of an early task (develop and harmonize protocols for single cell, spatial transcriptomic analysis of these tissues), and was similarly translated into a novel shared goal that consortium members collaborated towards achieving. These examples highlight the flexibility and power of Thompson's solution-based blueprint for identifying team needs on the go, adding tasks, tools, and solutions that may benefit the entire scientific community to accelerate research.

In the following sections, we describe how we developed and implemented this blueprint for the RE-JOIN consortium, and provide tips and resources for establishing the various elements needed for effective team science frameworks.

### 3.3 Define collaborative goals

An essential step during a team science project's development phase is to establish collaborative goals that are challenging, but achievable (23, 26). For projects starting with a team that organically assembled around a shared research question, this process may have largely taken place during team formation. In contrast, if a team is assembled by funding sources as a “team of research groups that are all interested in a similar research topic”, this process can be more challenging. Regardless of the team's makeup, developing shared, collaborative goals can be accomplished in a stepwise approach. Starting from the team's overarching goal (e.g., understanding joint pain), compare each member or subgroup's individual experimental goals to identify commonalities and differences. Depending on the project's nature and structure, an overarching goal may be seeded by the project's funding sources. However, each research group will have translated this goal into an individualized research plan based on its expertise and strengths. At the onset of a new collaboration, it is therefore important to explore the strengths, skills, and individual research goals that each subgroup and member brings to the table so that they can be integrated into a collaborative research plan with shared, achievable goals for the entire team.

**Figure 5:**
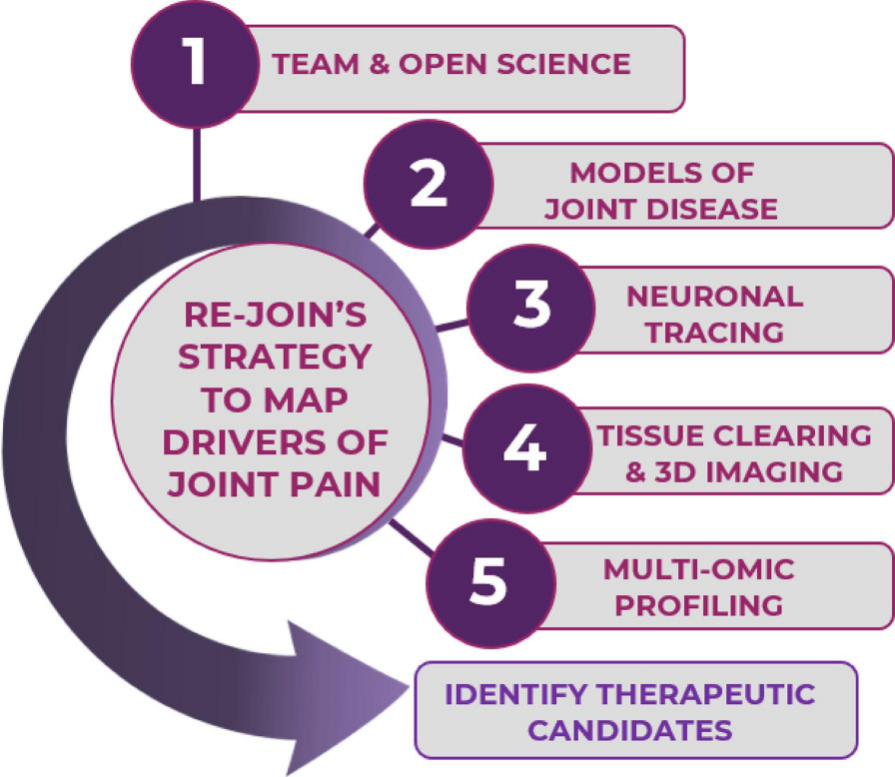
RE-JOIN's collaborative research goals include taking a team and open science approach to optimize and harmonize technologies, disease models, and to study chronic joint pain, aiming to identify joint disease-induced innervation changes responsible for patient pain experiences to identify novel therapeutic candidates and develop effective pain management strategies.

To develop RE-JOIN's shared goals, members joined an in-person kick-off meeting in Bethesda, Maryland to meet one another and discover each subgroup's research goals, skillsets, and expertise. We next discussed existing knowledge/technology gaps that hamper the study of chronic joint pain: reliable methods to label, visualize, and characterize joint-innervating neurons and their circuits, to assess distinct pain types across species, and to accurately model various types of joint disease across species. Finally, we discussed the tasks, tools, and outputs needed to fill these knowledge/technology gaps, and came up with a set of shared, collaborative goals to develop these elements (Figures 3, 5, Supplementary File S1). However, it is important to note that, like most other team science elements, collaborative goals will likely evolve throughout the project, so it is important to maintain a nimble attitude and adjust them as needed, without losing track of the overarching goal.

### 3.4 Establish trust and a collaborative mindset

Establishing and maintaining trust among team members is a critical aspect of any collaboration, especially for those initiating from multiple, competitively funded projects. Trust building initiates the moment team members meet. For RE-JOIN members, the in-person kick-off meeting provided an instrumental and productive start, but it took consortium members most of the first year to fully grasp each other's projects, scientific expertise and strengths. Scheduled consortium-wide presentations by team members combined with in-person and virtual unstructured interaction opportunities helped members get to know one another and build rapport (14, 26).

A culture of trust is also fostered by a responsible, transparent information sharing and decision-making process that values and incorporates team member input (12). To this end, RE-JOIN elected to open its meetings to all consortium members. In addition, we rely on Robert's rules of order for decision-making, with consortium-wide discussions that invite broad input prior to voting. Finally, detailed meeting minutes that contain clearly defined action items, deadlines, and assignees are circulated after each meeting to harness accountability and progress.

Researcher buy-in and trust also requires up-front discussions and agreements about how roles and responsibilities are divided, and how authorship will be handled (30). To build and maintain trust, it is critical to establish consortium values that appropriately recognize the power of diverse approaches and models in the preclinical domain with the need to harmonize and standardize data collection and sharing throughout the translational spectrum of science. In the case of RE-JOIN, discussions about responsibilities, roles, and credit were held early on, during the kick-off meeting, and were formalized into policies within the first few months. As part of the consortium's publication policy, the team decided to include a consortium authorship banner to the author list of consortium-related publications. RE-JOIN's banner lists all members who generate data and/or contribute intellectually to RE-JOIN's collaborative research goals, including trainees, research assistants, and early career researchers. This banner will be indexed and searchable on Pubmed, depending on journal policies. In addition, all banner members will be listed in the manuscript's acknowledgment section. Individuals responsible for data generation and/or writing of a manuscript will be individually listed as authors, while consortium members who helped build RE-JOIN's research framework will be grouped under the banner. This process ensures everyone receives appropriate credit for their efforts, bolstering a culture of trust.

### 3.5 Establish an organizational structure

The success of a transdisciplinary team science project hinges on inter- and intra-team collaborations to develop and harmonize research methods, tissue collection, processing, and analysis pipelines so that data obtained by different teams can be integrated and analyzed as a whole. In addition, transparent study design and productive discussions on how individual elements fit into the overarching project are crucial for minimizing experimental overlap, while maximizing the generation of meaningful data and research resources that can be mined and used by the entire scientific community. To accomplish these goals, it is important to define a clear organizational structure at the project's onset, enabling subgroups [working groups (WGs), task forces (TF)] to execute smaller focused achievable goals while higher-level strategic discussions are held at the consortium-wide level (7, 31).

**Figure 6:**
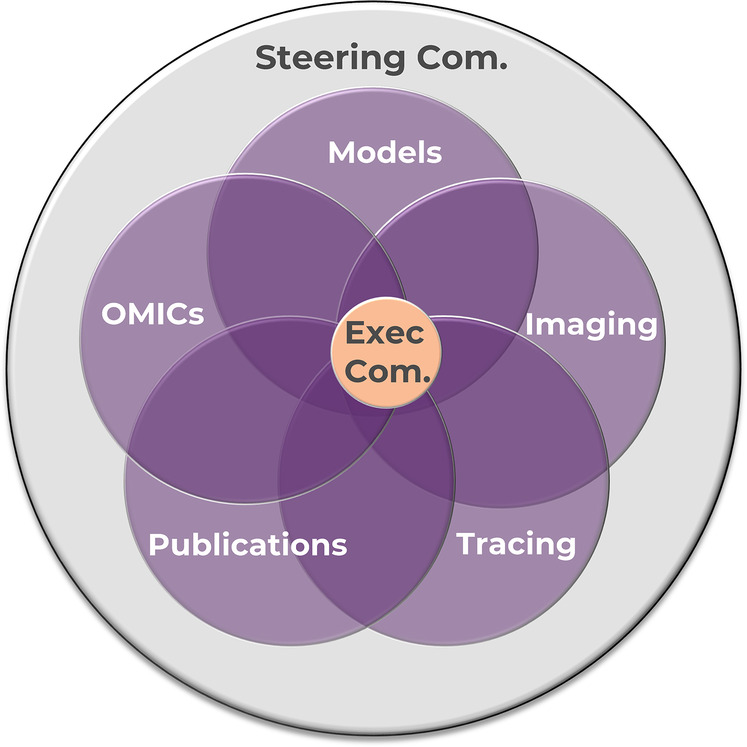
RE-JOIN consortium structure.

After comparing and discussing various organizational structures, RE-JOIN members established an executive committee, a steering committee, and multiple WGs (Figure 6). While individual WGs are tasked with developing a consortium-wide framework for a single discipline or joint type, overarching conversations aimed at integrating discipline-specific methods are held at consortium-wide meetings, such as the monthly steering committee and annual in-person RE-JOIN meetings. The steering committee (SC)'s main goal is to foster collaboration and knowledge exchange, outline collaborative research priorities, and implement the processes and working groups necessary to accomplish these goals. RE-JOIN's SC also functions as the consortium's main voting body to make strategic decisions and to achieve cross-project, transdisciplinary synergy. Prioritizing high-level discussions in inclusive, consortium-wide meetings bolsters team engagement and valuation because it promotes transparency, knowledge sharing, and rapport building among all team members, including trainees and early career researchers who are often overlooked for overarching, strategic conversations, and decision-making (7, 32). Hence, we opted for an inclusive approach, opening our SC meetings to all members to promote a culture of trust. In contrast, the executive committee (EC) consists of 1 representative per group, and is tasked with operationalizing the research agenda as set by the SC. In addition, the EC can convene *ad hoc* to make timely decisions on emerging items when needed. Finally, RE-JOIN's WGs are centered around methods and models to enable effective harmonization and alignment among member groups (Figure 6). Each WG is populated by topic experts (trainees, research assistants, postdocs, PIs), data scientists, team scientists, and NIH liaisons. Ensuring such broad WG membership will promote integration, innovation, and scientific progress (4), while ensuring the next generation of researchers learns the ins and outs of team science and pain research.

Finally, as a team's needs and goals change over time, the alignment between a team science project's collaborative goals and its operational structure should be assessed on a regular basis and should be adjusted as needed. To this end, the admin core annually surveys all members to identify any shifts in the consortium's operational needs. Our assessments provided the information needed for adjusting working groups, and led to the creation of additional task forces when additional needs (frameworks, standards, scoring methods, etc.) were identified over the years.

### 3.6 Build an operational infrastructure

Recent SciTS studies have emphasized the need for effective communication, knowledge sharing, and information exchange to promote successful team science (23, 26). Indeed, a lack of comprehensive information management strategies hinders development of strong team processes such as effective cross-communication, scientific coordination, and project management (12). Hence, team science endeavors should invest significant effort into developing information management toolkits for exchanging knowledge, information, data, files, code as early in the project as possible, as this will promote cross-consortium coordination and transparency. However, when building such toolkits, it is important to realize that personal and institutional preferences make it nearly impossible to identify an information sharing platform that works for everyone.

For example, RE-JOIN's information management toolkit consists of platforms to share files (Box), methods (protocols.io), data (SPARC/Pennsieve) and code (github). In addition, internal communication and coordination is fostered by information technology for email, calendar invites, and virtual Zoom or Teams meetings. We identified several strategies to maximize accessibility for all members:

– As adoption rates are higher for platforms that researchers already know, we minimized the number of unfamiliar platforms: the admin core first surveyed members to learn about (1) the platforms they know and (2) those they are allowed to use at their respective institutions. We subsequently built out RE-JOIN's information management strategy by incorporating elements familiar and accessible to all members where possible. The only element we did not find a universal platform for was file sharing: while nearly all members are able to use Box, 1 member institution restricts its use. To overcome this issue, we established parallel options for members to find, access, or share information.
– We developed a centralized RE-JOIN information hub on a web-based platform that is accessible to *all* pain researchers. The PURPOSE platform, supported by the HEAL® initiative, was recently created to connect pain researchers and promote communication and collaboration across and beyond HEAL-funded projects and consortia (20). RE-JOIN's private group page on PURPOSE contains general information about the consortium, its goals, policies, and information and data-sharing strategies. In addition, the page contains soft copies as well as links to the original files on Box, protocols.io, and others. Combined, this centralized access point lowers barriers for members to find information related to the consortium and supports efficient onboarding of new team members. Because the PURPOSE platform connects researchers across the pain and addiction spectrum (Figure 3), familiarizing RE-JOIN's members with the platform served a simultaneous purpose of promoting interaction and collaboration with other HEAL-funded initiatives.
– The admin core developed step-by-step manuals to guide members through using unfamiliar platform and included who to contact for help. These documents are all included on the consortium's PURPOSE page. In addition, the admin core and DCG offer various types of ongoing support, ranging from consortium-wide reminders to personalized one-on-one support.
– Finally, the admin core solicits frequent feedback and input on the consortium's information management toolkit, in the form of surveys, and makes adjustments where needed. For example, we adjusted RE-JOIN's information sharing workflow following members’ input that they were unable to access certain platforms (Box) due to institutional restrictions.

Efficient data sharing within and outside of the RE-JOIN consortium is supported by the consortium's data center (data coordinating group; DCG). As a HEAL-funded initiative, RE-JOIN investigators will comply with HEAL®'s public access and data sharing policy (33), and will make all meaningful primary data supporting its publications part of the HEAL Data Ecosystem by publishing their datasets on the SPARC (34), which leverages the Pennsieve data-sharing platform (34). In addition, the DCG helps ensure that all published RE-JOIN datasets are accompanied by the necessary metadata, protocols, and code to make them findable, accessible, interoperative, and reusable (FAIR) (35).

### 3.7 Create a data integration framework

Creating large, harmonized datasets that can be integrated across projects and initiatives to yield breakthrough discoveries is the aim of most team science endeavors. However, doing so requires significant effort from all team members, and involves creating various elements and standards that make up the initiative's data harmonization framework (27, 36). Such frameworks typically include:

– A *shared language* that bridges all disciplines involved in the endeavor to ensure discipline-specific jargon is used consistently and understood by all involved. For example, for RE-JOIN, this included developing a common anatomical vocabulary for all tissues that make up the knee and TMJ, including specific anatomical landmarks that define the tissue.
– *Harmonized study protocols and methods*, so that all studies are conducted as similarly as possible, save from experimental variables. For example, RE-JOIN investigators decided to harmonize sample collection and processing, and data analysis pipelines while allowing for some level of divergence among experimental paradigms, as this will permit comparing research results obtained from different teams while testing their robustness and validity through cross-comparisons (27). However, WGs performed in-depth comparisons of team-specific methods and protocols, shared through protocols.io, and aligned methods where possible to minimize variability.
– *Common data elements (CDEs) and (meta)data standards* that ensure consistent data element collection fashion across studies. CDEs are pre-determined data elements that have a set of controlled values (37, 38). When research projects adopt CDEs and data standards, resulting datasets contain consistent parameters and data elements, enabling seamless data integration. RE-JOIN'ers invested significant effort early in their project to develop its CDEs and data standards, which will enable consistent data collection across its basic and translational studies (27, 29). Because complying with data standards and CDEs typically represent a significant additional time investment for researchers, data and team science coordinating centers should create supporting documents—data dictionaries, (meta)data entry templates, etc.—where possible, as this can significantly lighten the load and reduce barriers for adoption (27, 36, 39). For example, we created a REDCap-based data dictionary to support HEAL and joint-pain-specific CDE clinical data collection for RE-JOIN's translational studies (29), and created metadata entry sheets and data dictionaries to support data collection for the consortium's basic studies (27, 28).
– *Data sharing and material transfer agreements* will allow internal sharing of data and resources as they are generated and will ensure appropriate intellectual property protections are in place. Depending on the consortium's nature and its goals, members can opt for generating separate agreements between member institutions to cover data or resource sharing as needed. Alternatively, the consortium can adopt consortium-wide agreements that cover both data and material transfers prior to publication. While it will likely take significant effort to generate such consortium-wide agreements, they will remove administrative barriers for sharing and will greatly facilitate collaboration among teams.
– *Common data analysis pipelines* to allow data, collected by decentralized teams, can be processed and analyzed using the same programs, algorithms, codes, and parameters. For example, RE-JOIN's Omics WG is developing a common bulk RNAseq pipeline that will be hosted on their internal data sharing platform, Pennsieve (40). Importantly, while these common pipelines enable consistent analysis of generated datasets for integration, individual research groups can still opt for using their own pipelines to interrogate individual datasets and pursue member-specific research questions.
– *Infrastructure and platforms* for internal and public sharing of these research resources. RE-JOIN's sharing platforms include protocols.io (methods), REDCap (data elements), Github (codes), Pennsieve (pilot datasets, common analytical pipelines), and SPARC (complete datasets).

Discussing data harmonization and standardization to establish a team science initiative's data harmonization framework during the project's early phase, when individual teams may still be finalizing their study and experimental design, is challenging. However, it is important to start this process prior to the start of data collection at scale, as this can significantly reduce data wrangling revisions for later analysis and data submission. Fortunately, several guides were recently published that help researchers navigate their data harmonization journeys and that include supporting resources to minimize the load (27, 36, 39, 41).

### 3.8 Embrace change to overcome challenges

The translation of ideas from discovery to clinical application is essential to advance effective therapeutic approaches. Thus, effective translational science is inarguably linked to effective team science. As shown through many examples above, developing and maintaining the elements that enable effective, solution-oriented team science requires a nimble attitude. To this end, team science initiatives will greatly benefit from building in regular moments of reflection, be it through surveys, small or large-group discussions, or *ad hoc* conversations. Such introspective conversations should combinedly cover all aspects of the supportive team science framework (Figure 1), and will help identify novel needs, tasks, tools, and outputs to further improve the consortium's efficacy and overall performance (Figure 3). In addition, these discussions help set the team up for success because they leverage collaborative problem solving to improve team communication and management while reinforcing a culture of trust and affect (8). For example, RE-JOIN's admin core distributes a multi-faceted annual survey to assess the performance of the consortium, its working groups, and its task forces. The results of these surveys are presented during the annual meeting, followed by presentations from WG leaders to update the consortium about their progress. We then hold group discussions to determine if any changes to the consortium's structure or operations are needed. In addition, we use this moment of reflection to assess the progress we have made towards our shared goals, and we outline deliverables and goals for the following year.

Despite careful planning and coordination, the nature of merging multiple teams and disciplines to achieve a shared goal will inadvertently be accompanied by challenges that emerge along the way. Some of these may relate to conflict, resulting from task-related or interpersonal disagreements (26, 31). In such situations, incorporating evidence-based strategies to prevent and resolve conflicts, which have been described in detail previously (42), will be essential to maintain team effectiveness.

A second type of team science-related challenge is to balance consortium-focused efforts (centripetal force) and team-focused efforts (centrifugal force) throughout the project's lifespan. This is especially true for consortia in which multiple research teams are competitively funded to achieve similar goals, like RE-JOIN. In such scenarios, there may be some correlation between the project's funding lifecycle and the magnitude of centripetal force: while members will be very invested in contributing to team science-focused efforts early on, the need to deliver on milestones that were included in their own grant applications may require them to divert effort away from shared team goals towards their individual project goals. In RE-JOIN's case, NIH leadership anticipated this challenge and included a requirement for consortium members to develop and initiate a set of collaborative projects in the last 2 years of the funding cycle. We found that this requirement stimulated teams to come together around ways to bolster innovation and impact, which helped them rebalance centripetal and centrifugal efforts at a crucial time in the project's lifecycle.

Lastly, an ongoing challenge for RE-JOIN members is to navigate limitations and regulations around data sharing. While it is our goal to share “hot off the press” data to accelerate collaboration and integration, we have encountered significant delays related to data sharing usage agreements (DUAs). The SC initially decided to establish individual DUAs between institutions as needed but have since revised that approach and are developing a consortium-wide agreement. However, differences in institutional and international data sharing policies combined with the sensitive nature of protected health information (PHI) human subject data make it very challenging to establish a consortium-wide DUA. We would advise other team science initiatives to start this process as early as possible to minimize administrative delays in collaborations. To conclude, here, we leveraged evidence-based conceptual models from the SciTS field to outline a practical roadmap for designing and implementing solution-oriented team science initiatives based on practical lessons learned from establishing the RE-JOIN. While the tips, resources, and examples we included here relate to studies in the pain and addiction field, they can be leveraged to support any transdisciplinary team science initiative on its path to breakthrough discoveries.

## Supplementary material

Supplementary Data Sheet 1 RE-JOIN's shared goals.

Supplementary Data Sheet 2 RE-JOIN teams and expertise.
