# Supplementary material for: Designing and implementing solution-oriented team science initiatives—a chronic pain example
Source: Front Pain Res (Lausanne). 2025 Dec 16;6:1669072. doi: 10.3389/fpain.2025.1669072 (PMC12748165; doi:10.3389/fpain.2025.1669072)
Supplement: Supplementary Data Sheet 1 — RE-JOIN's shared goals. [file Datasheet1.pdf]

Team Science Roadmap – Supplemental File: RE-JOIN's Collaborative Research Goals:

i. **Adopt Team Science and Open Science Principles**.
Solving the complex health problem of chronic joint pain will require a transdisciplinary approach that integrates insights from various scientific fields to collaboratively develop novel methods, resources, and tools to study neuronal innervation of joint-associated tissues. Rapid scientific advances in this field will depend on several key factors, including open sharing of optimized protocols and generated data, as well as harmonization of study designs and analysis pipelines. RE-JOIN members will develop minimal (meta)data standards and leverage existing platforms for open sharing of data meeting FAIR principles (SPARC), methods (protocols.io), and analysis pipelines (Github) within the consortium and beyond.

ii. **Harmonize and align human and animal studies of joint disease**.
The consortium brought preclinical and clinical joint disease experts together during the planning stages of the studies. This provides the unique opportunity to align aspects of patient studies, such as pain questionnaires, quantitative sensory testing, and biopsy collection, with relevant counterparts in animal studies. In addition, diverse joint disease models studied by RE-JOIN's research teams include spontaneous, genetic, and post-traumatic models of knee or TMJ disease in distinct animal models (mouse, rat, goat, primate, horse, etc.). Combined, these animal models enable consortium members to study and model distinct aspects of the symptomatic and histopathological spectrum observed in patients with joint disease. While consortium members initially focused on aligning animal models of joint disease across member groups, we quickly realized that this diversity was a strength rather than a weakness as it will enable validating findings across models to identify generalized versus disease-specific changes.

iii. **Adapt cutting-edge neuronal tracing technologies for use in musculoskeletal tissues.**
Neuronal tracing methods have revealed the intricate connectivity patterns of distinct subtypes of neurons in the central nervous system of several model organisms. Researchers have successfully adopted neuronal tracing protocols to study peripheral organ innervation, but their use in mapping neuronal dynamics and connectivity patterns in musculoskeletal tissues has remained quite limited. RE-JOIN investigators will evaluate, modify, and combine existing genetic, viral, and conventional tracing tools to develop a neuronal tracing protocol that is optimized for use in the knee and temporomandibular joints.

iv. **Optimize tissue clearing and 3D imaging technologies**.
Visualizing and analyzing a sensory neuron's path and connections from the joint-associated tissue it innervates to its cell body in the dorsal root or trigeminal ganglion will require the adoption and optimization of tissue clearing and 3D-imaging technologies for the distinct tissue types that make up the joint. Because optimal parameters to clear mineralized joint tissues will differ from those to clear peri-articular tissues including tendons, ligaments, synovia, or fat, RE-JOIN's imaging experts will have to balance competing requirements of collection and downstream analyses needs to efficiently clear the entire joint unit.

v. **Develop multi-omic maps of the joint, sensory, and autonomic ganglia**.
Apart from technologies to label and visualize various joint-innervating neuronal subtypes,

multi-omic analyses will be essential to identify molecular changes at the tissue and single cell level. RE-JOIN's planned approaches include bulk RNA sequencing, single cell RNA sequencing, ATACseq, as well as spatial transcriptomic and metabolomic analyses. As an important first step, consortium members will compare and harmonize methods to collect, process, and analyze data obtained from various relevant tissue types such as the synovium, dorsal root ganglion (DRG), trigeminal ganglion (TG), and others. This alignment will enable multi OMIC analyses and comparisons across tissue types, joint disease models, and joint types that may reveal molecular or cellular changes that mediate pain in knee and temporomandibular joints.

vi. **Identify therapeutic target candidates and responses to interventions.**
RE-JOIN's overarching goal is to develop an integrated 3-dimensional map of the knee and temporomandibular joints that reveal how joint innervation changes in function of biological sex, exercise, age, or disease. As part of this project, we will identify cellular and molecular differences between healthy and painful joints that may represent promising therapeutic candidates and evaluate how the newly discovered determinants of pain respond to existing interventions. While a therapeutic focus was not set as one of RE-JOIN's main priorities, it represents the ultimate goal of all our studies.
