# Supplementary material for: Designing and implementing solution-oriented team science initiatives—a chronic pain example
Source: Front Pain Res (Lausanne). 2025 Dec 16;6:1669072. doi: 10.3389/fpain.2025.1669072 (PMC12748165; doi:10.3389/fpain.2025.1669072)
Supplement: Supplementary Data Sheet 2 — RE-JOIN teams and expertise. [file Datasheet2.pdf]

Team	Institutions	Expertise	Clinical Research	Preclinical Research
**A-TMJ**<br>(18 members)	UTHSC at San Antonio<br>Karolinska Institute<br>University of Texas (UT) at San Antonio<br>UT Medical Branch at Galveston<br>Wake Forest Medical School	TMJ	TMJ disorders<br>patient surveys and evaluations<br>quantitative sensory testing<br>Multi-omics<br>innervation studies	animal models of TMD<br>acute and chronic pain<br>scRNAseq<br>neuronal tracing<br>electrophysiology<br>neuronal activity analysis<br>bioinformatics
**Blue**<br>(31 members)	Duke University<br>University of Michigan	TMJ	TMJ disorders<br>patient surveys and evaluations<br>quantitative sensory testing<br>Multi-omics<br>innervation studies	animal models of TMD<br>behavioral analysis<br>neuronal tracing<br>neuronal circuits<br>tissue clearing<br>3D Imaging<br>functional neuronal imaging<br>imaging and computational tool development
**UF-Pitt**<br>(34 members)	University of Florida<br>Pittsburgh University<br>Emory University<br>Emory University	TMJ<br>Knee Joint	TMJ disorders<br>Knee osteoarthritis<br>patient surveys and evaluations<br>quantitative sensory testing<br>innervation studies	animal models of TMD<br>animal models of OA<br>behavioral analysis of pain<br>electrophysiology<br>neuronal activity analysis<br>tissue clearing<br>3D Imaging
**kNERVE**<br>(30 members)	Baylor College of Medicine<br>University of Virginia<br>University of California, Irvine	Knee Joint	/	animal models of OA<br>behavioral analysis of pain<br>snRNAseq<br>spatial transcriptomics<br>tissue clearing<br>3D Imaging<br>neuronal tracing<br>neuronal circuits<br>gene therapy<br>bioinformatics<br>imaging and computational tool development
**M-Knees**<br>(23 members)	Rockefeller University<br>Rush University<br>Scripps<br>University of Michigan<br>Hospital for Special Surgery<br>Northwestern University<br>University of Texas at Dallas	Knee Joint	Knee Osteoarthritis<br>patient surveys and evaluations<br>quantitative sensory testing<br>Multi-omics<br>spatial transcriptomics<br>innervation studies	animal models of OA<br>behavioral analysis of pain<br>scRNAseq<br>spatial transcriptomics<br>tissue clearing<br>3D Imaging<br>neuronal tracing<br>neuronal circuits<br>neuronal activitiy analysis<br>imaging and computational tool development
**DCG**<br>(10 members)	University of Pennsylvania<br>University of California, San Diego	Data Science<br>Knowledge Engineering<br>Software Development		
**Admin Core**<br>(3 members)	Baylor College of Medicine	Team Science<br>Project Coordination<br>Project Administration		
